# Occupational Cancer Mortality Trends in Brazil, 1990–2023

**DOI:** 10.3390/ijerph23020184

**Published:** 2026-01-31

**Authors:** Louise Moura de Rezende, Cristiane de Oliveira Novaes, Clara Soares Rosas, Lara Barbosa de Souza Moura Canas Lara, Vitor Augusto de Oliveira Fonseca, Raphael Mendonça Guimarães

**Affiliations:** 1 Graduate Program in Environment and Public Health, National School of Public Health, Oswaldo Cruz Foundation (FIOCRUZ), Avenida Brasil 4036, 10th Floor, Rio de Janeiro 21041-210, Brazil; louimr@gmail.com; 2 Medical Ecucation Institute, Estácio de Sá University, Rio de Janeiro 22640-102, Brazil; novaes.cristiane@gmail.com (C.d.O.N.); cr556135@gmail.com (C.S.R.); laramoura.c@gmail.com (L.B.d.S.M.C.L.); 3 Public Health Institute, Federal University of the State of Rio de Janeiro, Rio de Janeiro 21941-617, Brazil; 4 Graduate Program in Public Health, National School of Public Health, Oswaldo Cruz Foundation (FIOCRUZ), Avenida Brasil 4036, 10th Floor, Rio de Janeiro 21041-210, Brazil; vitoroliveira_rj@hotmail.com

**Keywords:** work-related cancer, neoplasm mortality, occupational exposure, health inequalities, occupational health surveillance

## Abstract

Objective: This study analyzes temporal trends in occupational cancer mortality in Brazil and its federative units from 1990 to 2023, focusing on regional and gender disparities. Methods: We conducted an ecological time-series analysis using data from the Global Burden of Disease (GBD) database. We included deaths from malignant neoplasms attributable to occupational exposures and calculated age-standardized mortality rates. We applied segmented regression with the Joinpoint Regression Program (version 5.4) to estimate the Annual Percent Change (APC) and Average Annual Percent Change (AAPC) for Brazil and its states, stratified by sex. Results: Occupational cancer mortality declined nationally (AAPC = −1.08; 95% CI: −1.37 to −0.85), with a more substantial decrease among men. Marked regional differences emerged: the South, Southeast, and Midwest regions showed consistent declines, while several states in the North and Northeast exhibited stable or rising rates, especially among women. Part of the observed recent decline coincided with the COVID-19 pandemic (2019–2023), suggesting potential underdiagnosis or underreporting. Conclusion: Brazil has experienced a national decline in occupational cancer mortality; however, regional and gender inequalities persist. Territorial, economic, and occupational contexts shape these differences. Strengthening surveillance systems, updating exposure registries, and developing policies sensitive to regional and gender disparities may contribute to improving occupational cancer prevention and control.

## 1. Introduction

Work-related cancer remains a largely overlooked issue on the public agenda, despite representing one of the most severe outcomes of occupational exposures [1]. In Brazil—a country marked by profound regional inequalities and heterogeneity across its productive sectors—addressing occupational diseases, particularly those with long latency periods such as cancer, requires active surveillance, targeted policies, and investments in prevention. Delayed diagnosis and a historical pattern of underreporting contribute to the persistent underestimation of this condition in both health data and public health responses [2].

According to the World Health Organization (WHO), approximately 19% of all cancers are related to environmental exposures, including the workplace, which represents the primary route of exposure to a wide range of chemicals classified as carcinogenic by the International Agency for Research on Cancer (IARC) [3,4,5]. Moreover, a growing body of evidence supports the association between occupational settings and specific cancer types. Recent estimates indicate that between 3% and 8% of all cancer cases are attributable to occupational exposures [6,7,8,9]. Hazards such as asbestos, silica, pesticides, polycyclic aromatic hydrocarbons (PAHs), and chlorinated solvents remain prevalent in various Brazilian work environments, particularly in the mining, construction, and intensive agriculture sectors [10]. Nonetheless, occupational health information and surveillance systems in Brazil exhibit structural weaknesses, which limit the ability to identify consistent temporal and spatial patterns [11].

The evolution of the occupational cancer burden is especially relevant in a context of ongoing transformation in the productive structure, characterized by new forms of labor, precarious employment relationships, and the expansion of informal occupations [12,13]. Simultaneously, epidemiological surveillance has improved its capacity to attribute specific risks to occupational exposures, enabling increasingly refined analyses based on databases such as the Global Burden of Disease (GBD) [14]. However, in the Brazilian context, only acute intoxication cases are typically recorded, while chronic exposures—which are strongly associated with long-term health effects—are rarely reported. Against this background, the present study analyzes trends in occupational cancer mortality in Brazil between 1990 and 2023, disaggregating results by sex and state to identify regional patterns, temporal variations, and potential implications for occupational health surveillance and prevention. Our central hypothesis is that the observed national decline may obscure underlying structural inequalities and emerging exposures, particularly in regions with lower regulatory and enforcement capacities.

## 2. Methods

We conducted a descriptive, retrospective time-series study based on secondary data on mortality due to neoplasms attributable to occupational exposures, obtained from the Global Burden of Disease (GBD) database for the period from 1990 to 2023. The target population included individuals of both sexes, across all age groups, residing in Brazil during the study period. For each year and state, as well as for the country, age-standardized mortality rates were calculated.

Regarding the conceptual definition of occupational cancer and its link to exposure during working-age, it is important to note that, although GBD occupational cancer estimates are derived from exposure histories accumulated over the life course, the attribution framework is conceptually linked to the working-age population. Accordingly, our analytical focus is on occupational exposure-related mortality, primarily reflecting risks accumulated during working ages. Therefore, the results should not be interpreted as representative of total population cancer mortality.

Temporal trends in mortality rates were assessed using Joinpoint regression analysis, performed with the Joinpoint Regression Program, version 5.4.00 (NCI, Bethesda, MD, USA). In this model, the dependent variable was the age-standardized mortality rate, and the independent variable was the calendar year. The Joinpoint method enables the identification of statistically significant changes in time-series trends by fitting a segmented regression model that accounts for inflection points (“joinpoints”) at which changes in the trend pattern occur [15].

Mathematically, the analysis is based on fitting models of the following form:

$$\log\left(Y_{t}\right)=\beta_{0}+\beta_{1}t+\sum_{k=1}^{K}\delta_{k}{\left(t-\tau_{k}\right)}_{+}+\varepsilon_{t}$$

where:

- $Y_{t}$ is the age-standardized mortality rate at time ttt;
- t represents calendar year (time);
- $\beta_{0}$ is the model intercept;
- $\beta_{1}$ is the slope of the first segment;
- K is the number of joinpoints identified (up to four in this study);
- $\tau_{k}$ denotes the time at the k-th joinpoint;
- ${\left(t-\tau_{k}\right)}_{+}$ equals $t-\tau_{k}$ if t > $\tau_{k}$, and zero otherwise;
- $\delta_{k}$ represents the change in slope from the k-th joinpoint onward;
- $\varepsilon_{t}$ is the random error term, assumed to be homoscedastic and uncorrelated.

We assessed temporal trends in age-standardized mortality rates using a log-linear segmented regression approach. For each series (states × sex), we fitted models to the natural logarithm of the rate with 0, 1, or 2 joinpoints, enforcing a minimum of 4 observations per segment. We selected the best-fitting model using the Bayesian Information Criterion (BIC). We estimated models by ordinary least squares (OLS) under homoscedasticity assumptions for point estimation, while statistical inference (95% confidence intervals and *p*-values) relied on heteroskedasticity-consistent robust standard errors. For each fitted segment, we derived the Annual Percent Change (APC) as APC = $(e^{\beta }-1)\times 100$, where β is the segment-specific slope on the log scale. We also computed the Average Annual Percent Change (AAPC) for the entire period as a duration-weighted average of segment slopes (weights proportional to the number of years in each segment), reporting AAPC and 95% confidence intervals based on robust variance and the corresponding transformation to the percent scale [16].

The analysis was performed on a logarithmic scale, allowing for the estimation of Annual Percent Changes (APC) for each identified segment and the Average Annual Percent Change (AAPC) over the entire study period. The maximum number of joinpoints was limited to four, in accordance with methodological recommendations in the literature. Model parameters were estimated using the Ordinary Least Squares (OLS) method, under the assumption of uncorrelated random errors. We conducted a sensitive analysis prior to the first version of the manuscript to ensure that pandemic years did not affect the underlying time trends. Although we recognize that the COVID-19 pandemic may have distorted the magnitude of some health outcomes, such as cancer, the overall trend was not materially affected, given the availability of more than 30 years of pre-pandemic data. From a statistical perspective, it is unlikely that two or three observations would substantially alter an established long-term trend.

## 3. Results

**Figure 1:**
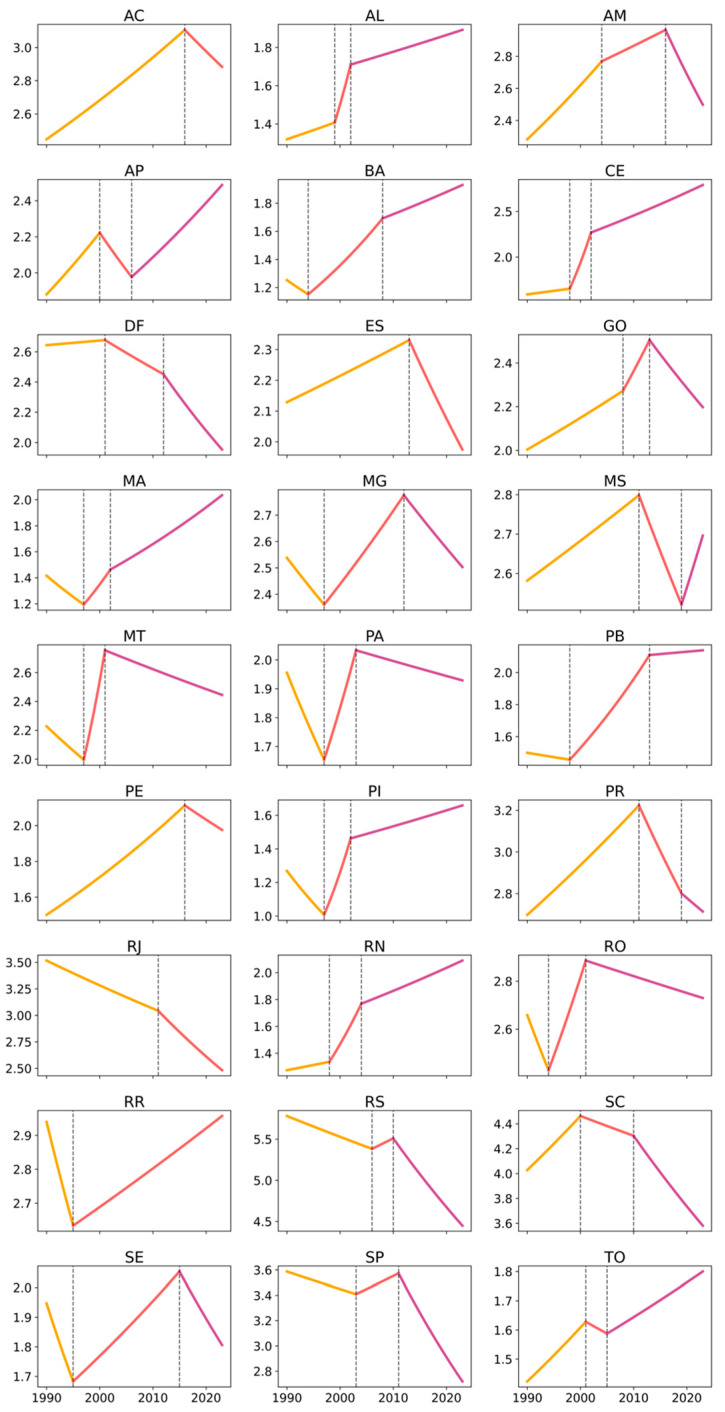
Age-standardized occupational cancer mortality trends (total population) by federative unit, Brazil, 1990–2023. Legend: Each panel corresponds to one federative unit, identified by its official two-letter abbreviation. Brazilian federative units are identified by their official two-letter abbreviations as follows: AC (Acre), AL (Alagoas), AP (Amapá), AM (Amazonas), BA (Bahia), CE (Ceará), DF (Federal District), ES (Espírito Santo), GO (Goiás), MA (Maranhão), MT (Mato Grosso), MS (Mato Grosso do Sul), MG (Minas Gerais), PA (Pará), PB (Paraíba), PR (Paraná), PE (Pernambuco), PI (Piauí), RJ (Rio de Janeiro), RN (Rio Grande do Norte), RS (Rio Grande do Sul), RO (Rondônia), RR (Roraima), SC (Santa Catarina), SP (São Paulo), SE (Sergipe), and TO (Tocantins). The x-axis represents calendar year (1990–2023), and the y-axis represents the age-standardized mortality rate (per 100,000 population). Solid colored lines indicate the fitted Joinpoint segments, with different colors representing distinct temporal segments. Vertical dashed lines mark statistically significant joinpoints, corresponding to years in which a significant change in trend slope occurred. Average Annual Percent Change (AAPC) estimates summarizing the overall trend for each state are reported in Table 1.

**Figure 2:**
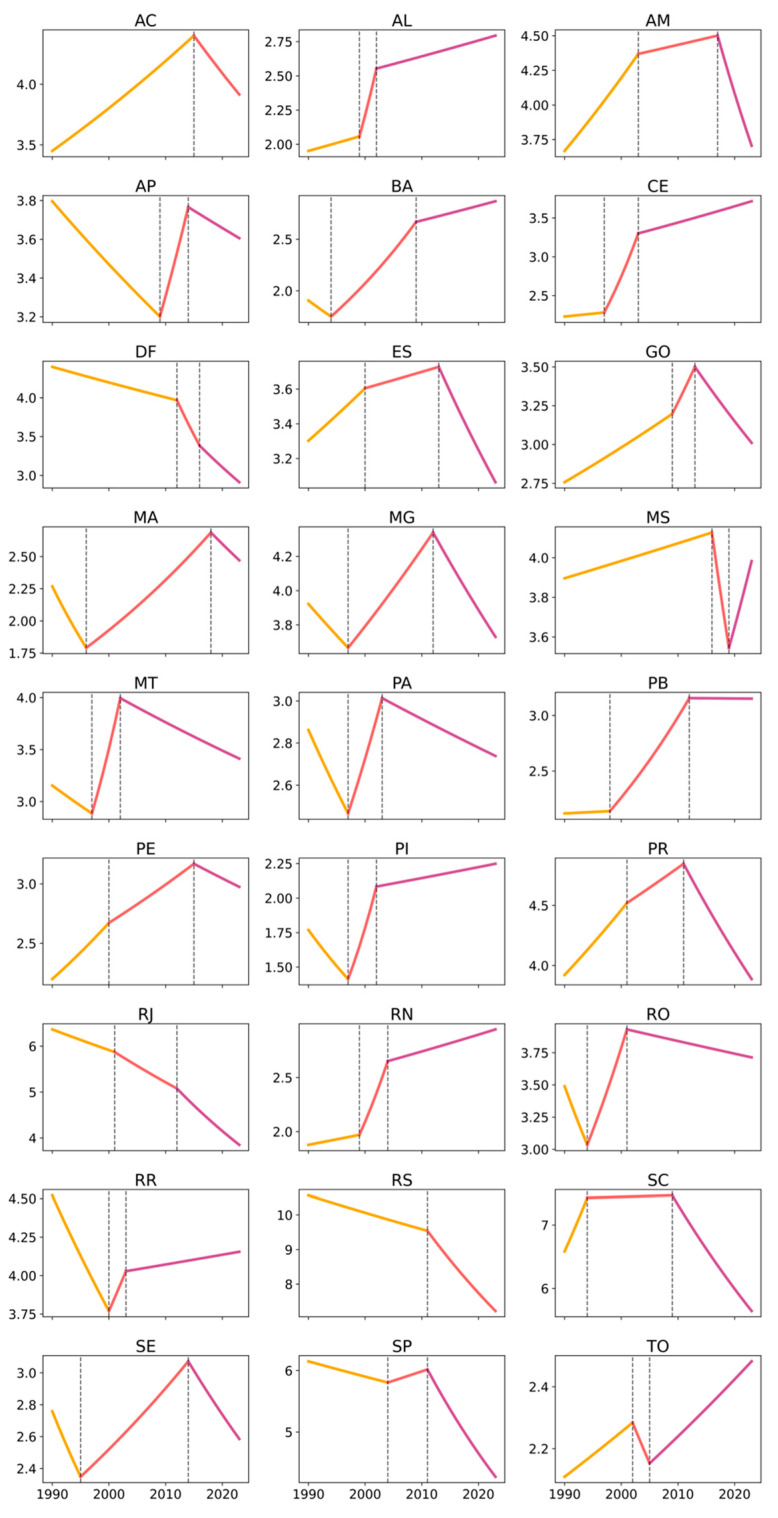
Age-standardized occupational cancer mortality trends among males by federative unit, Brazil, 1990–2023. Legend: Each panel corresponds to one federative unit, identified by its official two-letter abbreviation. Brazilian federative units are identified by their official two-letter abbreviations as follows: AC (Acre), AL (Alagoas), AP (Amapá), AM (Amazonas), BA (Bahia), CE (Ceará), DF (Federal District), ES (Espírito Santo), GO (Goiás), MA (Maranhão), MT (Mato Grosso), MS (Mato Grosso do Sul), MG (Minas Gerais), PA (Pará), PB (Paraíba), PR (Paraná), PE (Pernambuco), PI (Piauí), RJ (Rio de Janeiro), RN (Rio Grande do Norte), RS (Rio Grande do Sul), RO (Rondônia), RR (Roraima), SC (Santa Catarina), SP (São Paulo), SE (Sergipe), and TO (Tocantins). The x-axis shows calendar year (1990–2023), while the y-axis shows the age-standardized male occupational cancer mortality rate (per 100,000). Colored line segments represent the fitted Joinpoint trends, and vertical dashed lines denote statistically significant joinpoints.

**Figure 3:**
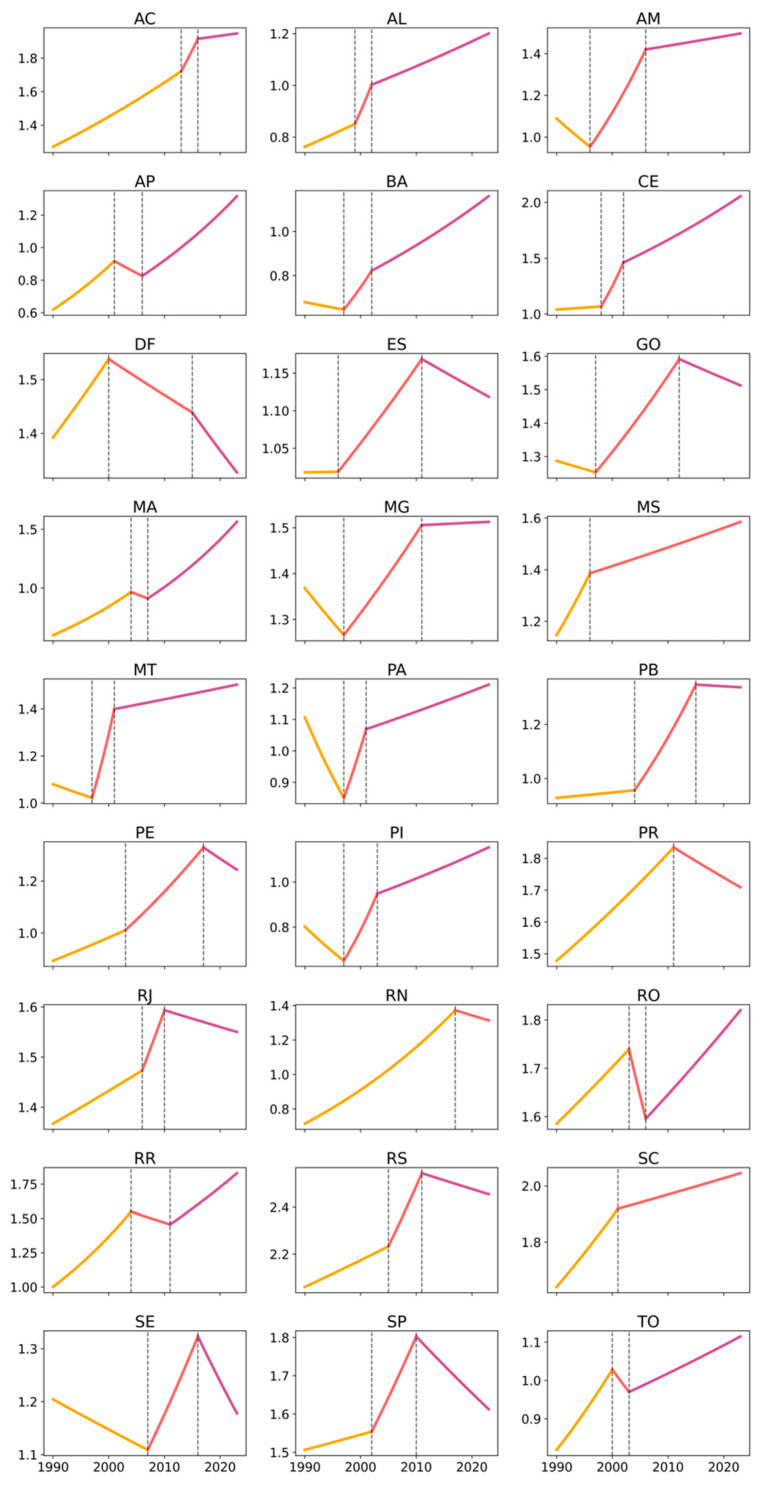
Age-standardized occupational cancer mortality trends among females by federative unit, Brazil, 1990–2023. Legend: Each panel corresponds to one federative unit, identified by its official two-letter abbreviation. Brazilian federative units are identified by their official two-letter abbreviations as follows: AC (Acre), AL (Alagoas), AP (Amapá), AM (Amazonas), BA (Bahia), CE (Ceará), DF (Federal District), ES (Espírito Santo), GO (Goiás), MA (Maranhão), MT (Mato Grosso), MS (Mato Grosso do Sul), MG (Minas Gerais), PA (Pará), PB (Paraíba), PR (Paraná), PE (Pernambuco), PI (Piauí), RJ (Rio de Janeiro), RN (Rio Grande do Norte), RS (Rio Grande do Sul), RO (Rondônia), RR (Roraima), SC (Santa Catarina), SP (São Paulo), SE (Sergipe), and TO (Tocantins). The x-axis shows calendar year (1990–2023), while the y-axis shows the age-standardized female occupational cancer mortality rate (per 100,000). Colored line segments represent the fitted Joinpoint trends, and vertical dashed lines denote statistically significant joinpoints.

Between 1990 and 2023, occupational cancer age-standardized mortality rates in Brazil showed an overall declining trend, particularly among men. Using Joinpoint regression with robust variance, the model identified an Average Annual Percent Change (AAPC) of −0.22% (95% CI: −0.29 to −0.15) for the total population, with a statistically significant decline among males (AAPC: −0.46%; 95% CI: −0.54 to −0.39), while female mortality increased at the national level (AAPC: 0.65%; 95% CI: 0.58 to 0.71). These findings indicate a persistent and widening sex differential in occupational cancer mortality trends. The analysis of temporal trends in age-standardized rates, assessed using the Joinpoint method, revealed both similarities and differences among Brazilian states in trend magnitude, direction, regional patterns, and sex-based disparities (Table 1; Figure 1, Figure 2 and Figure 3).

**Figure 4:**
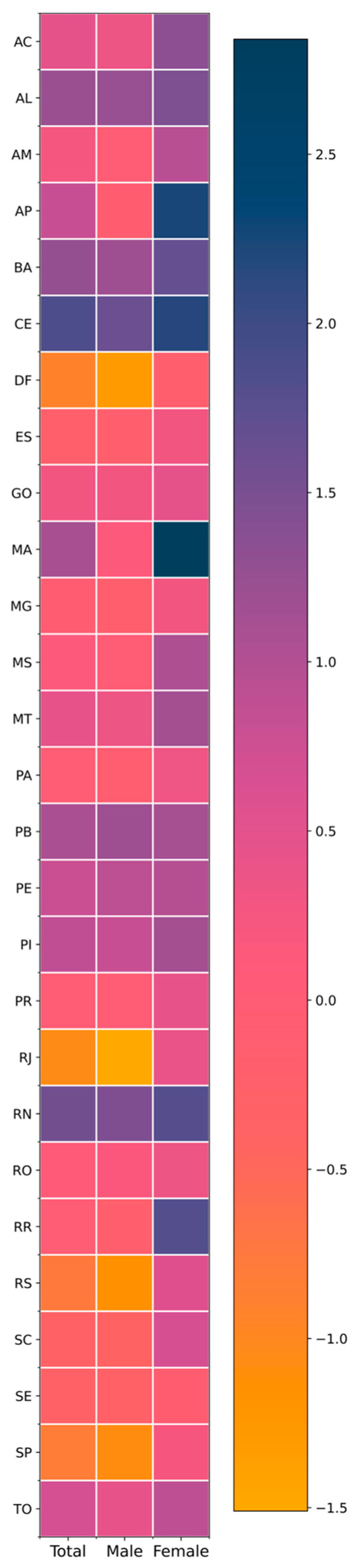
Average Annual Percent Change (AAPC) in age-standardized occupational cancer mortality by federative unit and sex, Brazil, 1990–2023. Legend: Each panel corresponds to one federative unit, identified by its official two-letter abbreviation. Brazilian federative units are identified by their official two-letter abbreviations as follows: AC (Acre), AL (Alagoas), AP (Amapá), AM (Amazonas), BA (Bahia), CE (Ceará), DF (Federal District), ES (Espírito Santo), GO (Goiás), MA (Maranhão), MT (Mato Grosso), MS (Mato Grosso do Sul), MG (Minas Gerais), PA (Pará), PB (Paraíba), PR (Paraná), PE (Pernambuco), PI (Piauí), RJ (Rio de Janeiro), RN (Rio Grande do Norte), RS (Rio Grande do Sul), RO (Rondônia), RR (Roraima), SC (Santa Catarina), SP (São Paulo), SE (Sergipe), and TO (Tocantins). Rows correspond to federative units, and columns correspond to sex strata. Cell color intensity reflects the magnitude and direction of the AAPC: warmer colors indicate increasing trends, and cooler colors indicate decreasing trends. AAPC estimates were derived from Joinpoint regression models with robust variance, accounting for multiple temporal segments where applicable. This figure provides a visual synthesis of regional and sex-based differences in trend magnitude reported numerically in Table 1.

Regarding trend magnitude (AAPC), most states in the North and Northeast exhibited positive average annual variations, often exceeding 1% per year, particularly among women. Notable examples include Amapá (women: 2.23% per year), Maranhão (2.84%), Ceará (2.19%), Rio Grande do Norte (1.80%), and Acre (1.34%), indicating accelerated increases in female occupational cancer mortality. These upward trajectories and their temporal segmentation are visually evident in Figure 3, which shows sustained growth phases and multiple inflection points for the female series in these states. Among men in these regions, positive trends were also observed in several states—such as Ceará (1.63%), Rio Grande do Norte (1.46%), Paraíba (1.19%), and Alagoas (1.23%)—although magnitudes were generally lower than those observed among women. This pattern is clearly illustrated in Figure 2 and Figure 4.

Conversely, states in the South and Southeast showed predominantly negative AAPCs among men and in the total population, reflecting stabilization or consistent declines in occupational cancer mortality. This pattern was particularly evident in São Paulo (men: −1.08%; total: −0.82%), Rio de Janeiro (men: −1.51%; total: −1.06%), Rio Grande do Sul (men: −1.16%; total: −0.77%), and Santa Catarina (men: −0.40%; total: −0.35%). Figure 1 and Figure 2 illustrate the relative linearity and long declining segments in these states, with few joinpoints and sustained negative slopes among men. Among women in these states, AAPCs were generally positive but modest, ranging from near zero to approximately 0.68%, suggesting relative stabilization rather than accelerated growth—a pattern that appears as lighter color gradients in Figure 4.

The Midwest region displayed an intermediate pattern: positive female trends were observed in Mato Grosso (1.13%) and Mato Grosso do Sul (1.04%). In contrast, male trends were negative in the Federal District (−1.32%) and close to null in Mato Grosso do Sul (0.01%). This intermediate positioning is visually reinforced in Figure 1, Figure 2 and Figure 3, where Midwest states alternate between patterns resembling the North/Northeast (female growth) and the South/Southeast (male decline).

Regarding trend direction, marked heterogeneity was evident across regions and sexes. In the North and Northeast, mortality rates increased consistently among women. At the same time, male trends were more varied, ranging from decline (e.g., Roraima) to sustained growth (e.g., Ceará, Rio Grande do Norte) or stability. Figure 2 and Figure 3 provide a side-by-side visualization of these divergent male and female trajectories within the same states, complementing the numerical contrasts shown in Table 1. In the South and Southeast, the dominant pattern was declining or stable male mortality, further reinforcing sex divergence in trajectories.

From a regional pattern perspective, three broad groupings emerged. The first, comprising the North and Northeast, was characterized by rising trends among women, positive or near-zero trends in the total population, and heterogeneous male patterns. The second, encompassing the Southeast and South, was marked by sustained declines among men, negative total AAPCs, and modest increases among women. The Midwest presented an intermediate profile. These regional groupings are synthesized visually in Figure 4, where clusters of higher AAPCs among women in the North/Northeast contrast sharply with negative male AAPCs in the South/Southeast.

The sex differential was striking nationwide. In 22 of the 27 states, women exhibited AAPCs equal to or greater than those of men. Clear examples include Rondônia, Mato Grosso do Sul, Rio de Janeiro, São Paulo, Rio Grande do Sul, and Santa Catarina, where female trends were positive or near zero while male trends were distinctly negative. Figure 4 highlights these contrasts through opposing color gradients by sex within the same state, reinforcing the magnitude of the differential described numerically in Table 1. At the national level, the sex gap reached 1.11 percentage points, confirming that recent increases in occupational cancer mortality are concentrated among women, while men experienced consistent reductions.

The time-series analysis using Joinpoint models further highlighted these disparities (Table 2; Figure 1, Figure 2 and Figure 3). In the North and Northeast, female time series were generally more fragmented, with multiple joinpoints indicating alternating periods of rapid growth, stabilization, and renewed increase. This higher temporal fragmentation among women is visually apparent in Figure 3, where several states display two or more inflection points. Among men, several states showed recent inflection points toward stabilization or decline, even after earlier growth phases (e.g., Maranhão, Paraíba, Pernambuco, Tocantins). In contrast, the South and Southeast displayed fewer joinpoints among men, with long segments of sustained decline (Figure 2), while female series often showed modest growth followed by stabilization (Figure 3).

At the national level, women experienced a prolonged period of growth between 1997 and 2012 (APC: 1.39%), followed by stabilization from 2012 onward (APC: −0.08%). Among men, a long period of near stability from 1990 to 1997 was followed by a modest increase until 2011, and then by a sharp and sustained decline from 2011 to 2023 (APC: −1.64%). These national trajectories are consistent with the patterns observed across most states in Figure 1, Figure 2 and Figure 3, confirming that women exhibit greater temporal fragmentation and larger magnitudes of change, while men tend to display greater linearity and sustained declines, particularly in more industrialized regions.

## 4. Discussion

Across the study period, occupational cancers accounted for a relatively small but non-negligible share of total cancer mortality in Brazil, representing approximately 2–4% of all cancer deaths, with higher proportions observed among men and in more industrialized regions [4,5]. This proportion remained broadly stable over time, despite divergent temporal trends by sex and region. The time-series analysis of age-standardized mortality across Brazil’s states, disaggregated by sex, reveals marked and systematic differences nationwide, in terms of magnitude, trend direction, and temporal stability. These patterns are particularly evident when comparing women and men across different regions, suggesting that epidemiological dynamics and the social determinants of mortality affect these groups differently [17].

The results of this study reveal a general trend of decreasing occupational cancer mortality in Brazil between 1990 and 2021, especially among men. However, this national trend, previously reported in other time-series studies [11], may conceal substantial disparities when regional and sex-specific variations are considered. In several states, distinct patterns emerged, with some regions showing stable or even increasing mortality rates, particularly among women. A central hypothesis emerging from these findings is that the observed national reductions reflect progress in regulating traditional occupational exposures—such as asbestos, benzene, and polycyclic aromatic hydrocarbons—in the most heavily regulated industrial sectors, especially in the South and Southeast. This dynamic aligns with recent literature indicating a global decline in the cancer burden attributable to occupational exposures in middle- and high-income countries, associated with substance substitution, improved working conditions, and stronger surveillance systems [18,19].

On the other hand, increasing or stable rates in specific regions and among women may be associated with the persistence of less regulated or emerging exposures, such as pesticides in agriculture, organic solvents in service sectors, and uncontrolled industrial waste in peripheral areas [20,21]. Pesticide exposure, for example, remains high in monoculture zones of the North, Northeast, and Midwest regions of Brazil. Another critical issue is the systemic underreporting of occupational exposures, which directly affects the accurate attribution of cancer-related deaths. Recent studies show that even in countries with more robust health systems, only a fraction of occupational cancer cases are correctly recorded in mortality data [22]. In Brazil, this gap is more severe for women, whose labor force participation is concentrated mainly in sectors historically overlooked by traditional occupational surveillance systems [23,24].

Furthermore, some of the observed declines coincide with the COVID-19 pandemic period (2019–2021), which may suggest underreporting of underlying causes of death, disruptions in diagnostic pathways, and interruptions in public surveillance policies [25,26]. These factors limit linear interpretations of the data and call for caution when drawing causal inferences. Moreover, regional analysis shows that states with stronger enforcement infrastructure—such as Rio Grande do Sul, São Paulo, and the Federal District—experienced more pronounced declines. In contrast, states in the North and Northeast, marked by high levels of informality, worker turnover, and weak social protection, exhibited irregular patterns, underscoring the importance of context-sensitive policies tailored to local productive and social realities [27,28].

The divergence we observed—rising female occupational cancer mortality alongside declining male mortality—is plausibly driven by a combination of gendered labor-market dynamics, differential exposure profiles, and structural surveillance limitations. Women’s employment has expanded and intensified in service, care, and cleaning occupations, where carcinogenic or potentially carcinogenic exposures may be underrecognized and undermeasured relative to traditional male-dominated industries; recent syntheses emphasize that women’s occupational cancer risks are often underestimated because exposure assessment, compensation frameworks, and research priorities historically center on “classic” industrial hazards and male job titles. In contrast, women’s tasks involve complex mixtures (e.g., disinfectants/solvents, sterilants, cytotoxic drugs, formaldehyde, and other agents in health and care settings) with incomplete exposure characterization and weaker regulatory visibility [29,30]. Moreover, women are disproportionately represented in shift-based care and service work. The IARC classification of night shift work as probably carcinogenic, together with ongoing epidemiologic linking it to breast cancer, provides a biologically plausible pathway for rising female burden in sectors with expanding night work, even as specific heavy industrial exposures among men decline due to regulation and industrial restructuring [31].

Finally, surveillance and exposure registries remain incomplete in many settings, and global occupational cancer burden work highlights that exposure prevalence data are often scarce, and that attribution is sensitive to gaps in measurement—limitations likely to be amplified for women’s heterogeneous jobs, mixed exposures, and underreported work histories [32]. Taken together, these dynamics support the interpretation that the apparent rise among women may reflect both real increases (or slower declines) in hazardous exposures in feminized sectors and historical systematic under-ascertainment. In contrast, declining male trends may reflect longer-standing reductions in high-intensity industrial carcinogen exposures and improved controls in regulated sectors. In this sense, the gender disparity, evidenced by rising or stagnant mortality rates among women in various states, should be interpreted as an epidemiological alert. Recent literature has highlighted female occupational exposure to carcinogens in sectors not traditionally monitored, such as retail, cleaning, healthcare, small-scale agriculture, and the textile industry [33,34]. These findings call for expansion of surveillance strategies to include underexplored occupational categories.

The findings from this time-series analysis of age-standardized mortality, disaggregated by sex and state, provide essential inputs for the critical evaluation and refinement of Brazil’s National Policy on Workers’ Health (PNSTT). The results clearly demonstrate that the trajectories of work-related cancer mortality in Brazil are not homogeneous. On the contrary, they are shaped by profound regional inequalities and substantial gender disparities, which both reflect—and challenge—the current foundations and strategies of public policy.

Formulated under the principles of equity and universality within the Brazilian Unified Health System (SUS), the PNSTT advocates intersectoral actions and health surveillance capable of addressing the country’s diverse realities [35]. However, the analysis of temporal trends reveals that implementation of these actions is uneven: for example, the North and Northeast regions show rising and unstable mortality rates among women, suggesting growing exposure to occupational risks or deficits in prevention, diagnosis, and reporting of work-related illnesses [24,27]. In the Southeast and South, declining or stable rates are observed among men. At the same time, women often exhibit rising or sustained mortality rates—a phenomenon that may reflect shifts in women’s labor force participation, with increasing entry into higher-risk or more precarious sectors, without commensurate expansion of protective policies [27,35].

These regional and gender differences are closely tied to Brazil’s social, technical, and gendered division of labor. Historically, women have been underrepresented in high-risk sectors such as heavy industry and construction. However, the data point to a transition: increased female presence in hazardous occupations, growing informality, and higher workloads due to double or triple work shifts [36], especially in the North and Northeast. Current public policy still tends to focus surveillance and prevention efforts on traditional sectors, often overlooking emerging risks and underestimating the impact of informal and precarious female labor. Furthermore, the persistence of regional inequalities suggests that national strategies have been insufficient to address local specificities, particularly in territories where access to occupational health services remains limited.

The disparities identified in mortality trends also call for a reevaluation of information and surveillance systems. Greater temporal fragmentation and trend instability among women, as well as abrupt reversals in some states, may reflect failures in data collection, underreporting of occupational diseases among women, or sudden changes in working conditions due to economic policies, crises, or regional productive restructuring. Within this context, the PNSTT must strengthen epidemiological surveillance mechanisms [17,28,37], particularly in historically vulnerable regions, and incorporate strategies to address the specific risks associated with informal and female labor.

Given the regional inequalities and sex-based differentials identified in this study, it is essential that the PNSTT advance on several fronts to ensure more effective and equitable responses across the national territory. First, actions and resources must be regionally adapted to recognize and respond to local diversity, especially in the North and Northeast, where mortality rates—particularly among women—are increasing and highly unstable. Funding and service organizations should promote coordination among occupational health, women’s health, and public health surveillance, prioritizing the most vulnerable areas.

Moreover, it is urgent to further integrate a gender perspective as a core axis of public policy. It involves identifying and monitoring the specific occupational risks faced by women, developing targeted education and training campaigns, and implementing protection strategies for informal, domestic, and emerging sectors that predominantly employ female labor. It also requires moving beyond the traditional view that occupational risks are confined to male-dominated sectors, by strengthening surveillance in “invisible” female labor segments, valuing mental health, and preventing harms related to harassment, overwork, and precarious employment—especially in reproductive health services and care work sectors. We emphasize that expanded cancer screening—particularly for breast and cervical cancer—may contribute to increased detection and certification of cancer deaths among women in certain regions, even though these cancers are not the most common work-related types of cancer. We also emphasize that screening alone is unlikely to fully explain the observed trends, given the occupational attribution framework and the consistency of increases across multiple states.

Another central point is improving information and surveillance systems. Investment in data quality, training for health professionals, and the integrated use of multiple databases are essential to enhance the identification of work-related illnesses, particularly among women and informal workers, and to capture emerging dynamics across other territories. Finally, strengthening mechanisms for social participation and intersectorality is key—engaging trade unions, social movements, and health councils so that workers’ lived experiences meaningfully inform the planning, implementation, and evaluation of public actions. In doing so, the PNSTT can become more responsive to regional realities and gender-specific conditions, driving concrete progress in reducing inequalities and promoting the health of all Brazilian workers.

This study has some limitations that must be considered when interpreting the results. First, the data source—based on Global Burden of Disease (GBD) estimates—uses global criteria for attributing occupational cancers, which may underestimate specific exposure contexts in Brazil. In addition, while the statistical modeling and correction techniques employed by GBD improve the quality and completeness of time-series data, they may also introduce biases inherent to the statistical adjustment process, particularly in countries with significant regional heterogeneity. To sum up, there are four key aspects that should be highlighted: (i) mortality data may have regional heterogeneity in quality and cancer attribution; (ii) there are inherent constraints of ecological designs, including the impossibility of individual-level inference; and (iii) there are potential distortions arising from underdiagnosis, delayed reporting, and uneven healthcare access and quality across regions; (iv) GBD estimates of occupational cancer do not control for individual-level confounders such as smoking, alcohol consumption, genetic susceptibility, lifestyle, and socioeconomic status. While GBD applies counterfactual modeling to estimate attributable fractions, residual confounding remains possible. Despite these limitations, using this database offers the advantage of enabling comparability across historical periods and regions, which aligns with the study’s primary objective of robust, and standardized trend analysis.

## 5. Conclusions

The overall declining trend in occupational cancer mortality in Brazil, observed between 1990 and 2021, represents a significant advancement in protecting workers’ health. However, this positive trend should not be interpreted as evidence of problem resolution. On the contrary, the data reveal significant disparities between regions and sexes, as well as indications of both persistent and emerging hazardous occupational exposures, particularly in contexts marked by informality, weak regulation, and institutional fragility. In this regard, strengthening occupational health surveillance is essential, including the expansion of information systems, investing in health workforce training, and the promotion of intersectoral coordination among health, labor, environmental, and justice sectors. It is also recommended that the list of carcinogenic substances of national concern be regularly updated, in line with the latest epidemiological and toxicological evidence.

The findings also indicate a partial convergence in mortality rates between men and women, especially in states with more advanced epidemiological surveillance, and underscore the need for differentiated approaches in public health policies that consider not only regional specificities but also the gender inequalities embedded in Brazil’s epidemiological profile. Ultimately, occupational cancer mortality should be understood as a marker of inequality and a failure of the preventive dimensions of the worker protection system. Despite relatively low absolute rates, it remains a preventable phenomenon with a high individual and societal burden, requiring a decisive and sustained response from health authorities and the public health field.

## Figures and Tables

**Table 1:** Average annual percentage change (AAPC) in the mortality rate from work-related cancers by sex. Brazil and State, 1990–2023.

Place	Male		Female		Total	
	AAPC (95% CI)	*p* Value	AAPC (95% CI)	*p* Value	AAPC (95% CI)	*p* Value
**North**						
Acre	0.35 (0.27 to 0.43)	<0.001	1.34 (1.26 to 1.41)	<0.001	0.47 (0.40 to 0.53)	<0.001
Amazonas	−0.02 (−0.18 to 0.14)	0.819	0.95 (0.82 to 1.07)	<0.001	0.24 (0.07 to 0.40)	0.006
Amapá	−0.09 (−0.29 to 0.11)	0.359	2.23 (2.07 to 2.40)	<0.001	0.81 (0.64 to 0.98)	<0.001
Pará	−0.1 (−0.22 to 0.01)	0.085	0.32 (0.20 to 0.44)	<0.001	−0.02 (−0.11 to 0.08)	0.731
Rondônia	0.17 (−0.35 to 0.70)	0.509	0.35 (0.22 to 0.47)	<0.001	0.07 (−0.36 to 0.51)	0.733
Roraima	−0.22 (−0.33 to −0.11)	<0.001	1.81 (1.54 to 2.08)	<0.001	−0.03 (−0.14 to 0.07)	0.509
Tocantins	0.44 (0.33 to 0.55)	<0.001	0.89 (0.79 to 0.98)	<0.001	0.69 (0.58 to 0.80)	<0.001
**Northeast**						
Alagoas	1.23 (1.11 to 1.35)	<0.001	1.48 (1.38 to 1.58)	<0.001	1.22 (1.11 to 1.32)	<0.001
Bahia	1.17 (1.08 to 1.27)	<0.001	1.66 (1.59 to 1.72)	<0.001	1.25 (1.18 to 1.32)	<0.001
Ceará	1.63 (1.53 to 1.73)	<0.001	2.19 (2.08 to 2.30)	<0.001	1.84 (1.76 to 1.93)	<0.001
Maranhão	0.13 (−0.02 to 0.29)	0.088	2.84 (2.67 to 3.01)	<0.001	1.10 (0.98 to 1.23)	<0.001
Paraíba	1.19 (1.09 to 1.29)	<0.001	1.11 (1.00 to 1.22)	<0.001	1.06 (0.96 to 1.15)	<0.001
Pernambuco	0.91 (0.80 to 1.01)	<0.001	0.98 (0.84 to 1.11)	<0.001	0.80 (0.71 to 0.88)	<0.001
Piauí	0.81 (0.71 to 0.90)	<0.001	1.14 (0.99 to 1.28)	<0.001	0.88 (0.78 to 0.98)	<0.001
Rio Grande do Norte	1.46 (1.31 to 1.60)	<0.001	1.80 (1.70 to 1.90)	<0.001	1.56 (1.40 to 1.72)	<0.001
Sergipe	−0.28 (−0.49 to −0.07)	0.010	−0.07 (−0.24 to 0.10)	0.430	−0.31 (−0.49 to −0.12)	0.002
**Southeast**						
Espírito Santo	−0.23 (−0.30 to −0.16)	<0.001	0.28 (0.16 to 0.40)	<0.001	−0.25 (−0.31 to −0.19)	<0.001
Minas Gerais	−0.17 (−0.32 to −0.02)	0.024	0.28 (0.22 to 0.35)	<0.001	−0.06 (−0.17 to 0.05)	0.255
Rio de Janeiro	−1.51 (−1.59 to −1.44)	<0.001	0.41 (0.35 to 0.47)	<0.001	−1.06 (−1.11 to −1.01)	<0.001
São Paulo	−1.08 (−1.17 to −0.99)	<0.001	0.22 (0.13 to 0.32)	<0.001	−0.82 (−0.91 to −0.74)	<0.001
**South**						
Paraná	−0.02 (−0.09 to 0.05)	0.575	0.43 (0.34 to 0.52)	<0.001	−0.03 (−0.10 to 0.04)	0.403
Rio Grande do Sul	−1.16 (−1.27 to −1.04)	<0.001	0.56 (0.43 to 0.68)	<0.001	−0.77 (−0.88 to −0.65)	<0.001
Santa Catarina	−0.40 (−0.63 to −0.17)	0.001	0.68 (0.58 to 0.78)	<0.001	−0.35 (−0.50 to −0.20)	<0.001
**Midwest**						
Distrito Federal	−1.32 (−1.45 to −1.19)	<0.001	−0.14 (−0.24 to −0.04)	0.006	−0.91 (−1.00 to −0.82)	<0.001
Goiás	0.29 (0.20 to 0.38)	<0.001	0.47 (0.32 to 0.62)	<0.001	0.29 (0.21 to 0.38)	<0.001
Mato Grosso do Sul	0.01 (−0.15 to 0.16)	0.925	1.04 (0.88 to 1.19)	<0.001	0.14 (0.01 to 0.27)	0.035
Mato Grosso	0.35 (0.12 to 0.57)	0.003	1.13 (1.03 to 1.23)	<0.001	0.43 (0.26 to 0.59)	<0.001
Brazil	−0.46 (−0.54 to −0.39)	<0.001	0.65 (0.58 to 0.71)	<0.001	−0.22 (−0.29 to −0.15)	<0.001

**Source:** GBD study, 2025.

**Table 2:** Temporal trend of mortality rate from work-related cancers by sex. Brazil and State, 1990–2023.

State	Male			Female		
	Period	APC (95% CI)	*p* Value	Period	APC (95% CI)	*p* Value
**North**						
Acre	1990–2015	0.98 (0.90 to 1.06)	<0.001	1990–2013	1.33 (1.20 to 1.46)	<0.001
	2015–2023	−1.45 (−1.81 to −1.09)	<0.001	2013–2016	3.62 (2.76 to 4.48)	<0.001
				2016–2023	0.24 (−0.12 to 0.60)	0.188
Amazonas	1990–2003	1.35 (0.94 to 1.77)	<0.001	1990–1996	−2.18 (−3.10 to −1.26)	<0.001
	2003–2017	0.21 (−0.03 to 0.46)	0.085	1996–2006	4.06 (3.58 to 4.54)	<0.001
	2017–2023	−3.19 (−3.72 to −2.66)	<0.001	2006–2023	0.31 (0.04 to 0.58)	0.025
Amapá	1990–2009	−0.89 (−1.28 to −0.51)	<0.001	1990–2001	3.64 (3.39 to 3.89)	<0.001
	2009–2014	3.30 (2.54 to 4.07)	<0.001	2001–2006	−2.08 (−2.80 to −1.36)	<0.001
	2014–2023	−0.48 (−1.05 to 0.09)	0.094	2006–2023	2.78 (2.32 to 3.23)	<0.001
Pará	1990–1997	−2.11 (−2.71 to −1.50)	<0.001	1990–1997	−3.69 (−4.29 to −3.07)	<0.001
	1997–2003	3.40 (2.8 to 4.00)	<0.001	1997–2001	5.88 (5.03 to 6.74)	<0.001
	2003–2023	−0.48 (−0.69 to −0.27)	<0.001	2001–2023	0.57 (0.37 to 0.76)	<0.001
Rondônia	1990–1994	−3.43 (−7.51 to 0.83)	0.109	1990–2003	0.72 (0.11 to 1.33)	0.023
	1994–2001	3.76 (2.75 to 4.79)	<0.001	2003–2006	−2.84 (−4.76 to −0.88)	0.006
	2001–2023	−0.26 (−0.47 to −0.05)	0.017	2006–2023	0.78 (0.59 to 0.96)	<0.001
Roraima	1990–2000	−1.80 (−2.12 to −1.48)	<0.001	1990–2004	3.17 (2.59 to 3.76)	<0.001
	2000–2003	2.23 (1.29 to 3.19)	<0.001	2004–2011	−0.88 (−1.46 to −0.29)	0.005
	2003–2023	0.15 (−0.06 to 0.37)	0.144	2011–2023	1.92 (1.23 to 2.62)	<0.001
Tocantins	1990–2002	0.66 (0.38 to 0.95)	<0.001	1990–2000	2.30 (1.91 to 2.70)	<0.001
	2002–2005	−1.95 (−2.70 to −1.19)	<0.001	2000–2003	−1.93 (−2.78 to −1.07)	<0.001
	2005–2023	0.79 (0.56 to 1.02)	<0.001	2003–2023	0.70 (0.52 to 0.88)	<0.001
**Northeast**						
Alagoas	1990–1999	0.59 (0.37 to 0.81)	<0.001	1990–1999	1.23 (0.96 to 1.50)	<0.001
	1999–2002	7.45 (6.24 to 8.68)	<0.001	1999–2002	5.60 (4.51 to 6.70)	<0.001
	2002–2023	0.43 (0.13 to 0.73)	0.007	2002–2023	0.86 (0.64 to 1.09)	<0.001
Bahia	1990–1994	−2.10 (−2.90 to −1.29)	<0.001	1990–1997	−0.72 (−0.97 to −0.47)	<0.001
	1994–2009	2.84 (2.68 to 3.01)	<0.001	1997–2002	4.94 (4.65 to 5.24)	<0.001
	2009–2023	0.52 (0.33 to 0.70)	<0.001	2002–2023	1.65 (1.54 to 1.76)	<0.001
Ceará	1990–1997	0.33 (−0.06 to 0.72)	0.095	1990–1998	0.33 (−0.18 to 0.85)	0.195
	1997–2003	6.35 (5.90 to 6.80)	<0.001	1998–2002	8.15 (7.26 to 9.05)	<0.001
	2003–2023	0.59 (0.42 to 0.76)	<0.001	2002–2023	1.64 (1.45 to 1.82)	<0.001
Maranhão	1990–1996	−3.86 (−4.78 to −2.93)	<0.001	1990–2004	3.47 (3.03 to 3.91)	<0.001
	1996–2018	1.86 (1.66 to 2.06)	<0.001	2004–2007	−1.93 (−3.45 to −0.38)	0.016
	2018–2023	−1.66 (−2.40 to −0.91)	<0.001	2007–2023	3.43 (3.03 to 3.83)	<0.001
Paraíba	1990–1998	0.12 (−0.11 to 0.35)	0.286	1990–2004	0.21 (0.01 to 0.42)	0.037
	1998–2012	2.82 (2.69 to 2.94)	<0.001	2004–2015	3.16 (2.91 to 3.41)	<0.001
	2012–2023	−0.01 (−0.33 to 0.31)	0.934	2015–2023	−0.09 (−0.59 to 0.41)	0.711
Pernambuco	1990–2000	1.97 (1.53 to 2.41)	<0.001	1990–2003	0.96 (0.81 to 1.10)	<0.001
	2000–2015	1.14 (0.88 to 1.41)	<0.001	2003–2017	1.98 (1.78 to 2.17)	<0.001
	2015–2023	−0.79 (−1.20 to −0.37)	0.001	2017–2023	−1.10 (−1.88 to −0.32)	0.007
Piauí	1990–1997	−3.16 (−3.64 to −2.67)	<0.001	1990–1997	−2.95 (−3.58 to −2.31)	<0.001
	1997–2002	8.08 (7.36 to 8.80)	<0.001	1997–2003	6.48 (6.00 to 6.97)	<0.001
	2002–2023	0.36 (0.20 to 0.53)	<0.001	2003–2023	0.99 (0.78 to 1.19)	<0.001
Rio Grande do Norte	1990–1999	0.54 (−0.03 to 1.11)	0.063	1990–2017	2.45 (2.33 to 2.56)	<0.001
	1999–2004	6.12 (5.11 to 7.15)	<0.001	2017–2023	−0.73 (−1.14 to −0.32)	0.001
	2004–2023	0.55 (0.27 to 0.84)	<0.001			
Sergipe	1990–1995	−3.17 (−4.30 to −2.03)	<0.001	1990–2007	−0.48 (−0.69 to −0.27)	<0.001
	1995–2014	1.42 (1.16 to 1.69)	<0.001	2007–2016	1.98 (1.64 to 2.32)	<0.001
	2014–2023	−1.90 (−2.60 to −1.19)	<0.001	2016–2023	−1.65 (−2.42 to −0.86)	<0.001
**Southeast**						
Espírito Santo	1990–2000	0.88 (0.59 to 1.17)	<0.001	1990–1996	0.01 (−0.78 to 0.81)	0.973
	2000–2013	0.26 (0.02 to 0.50)	0.036	1996–2011	0.92 (0.70 to 1.14)	<0.001
	2013–2023	−1.94 (−2.24 to −1.65)	<0.001	2011–2023	−0.36 (−0.56 to −0.17)	0.001
Minas Gerais	1990–1997	−0.96 (−1.70 to −0.21)	0.013	1990–1997	−1.11 (−1.37 to −0.84)	<0.001
	1997–2012	1.13 (0.98 to 1.29)	<0.001	1997–2011	1.25 (1.12 to 1.38)	<0.001
	2012–2023	−1.37 (−1.52 to −1.22)	<0.001	2011–2023	0.04 (−0.17 to 0.24)	0.696
Rio de Janeiro	1990–2001	−0.73 (−1.00 to −0.46)	<0.001	1990–2006	0.47 (0.36 to 0.58)	<0.001
	2001–2012	−1.32 (−1.53 to −1.11)	<0.001	2006–2010	1.98 (1.55 to 2.41)	<0.001
	2012–2023	−2.49 (−2.70 to −2.27)	<0.001	2010–2023	−0.21 (−0.39 to −0.03)	0.024
São Paulo	1990–2004	−0.41 (−0.66 to −0.16)	0.002	1990–2002	0.26 (−0.03 to 0.55)	0.076
	2004–2011	0.52 (0.16 to 0.88)	0.006	2002–2010	1.87 (1.62 to 2.13)	<0.001
	2011–2023	−2.82 (−3.01 to −2.62)	<0.001	2010–2023	−0.85 (−1.05 to −0.66)	<0.001
**South**						
Paraná	1990–2001	1.30 (1.14 to 1.46)	<0.001	1990–2011	1.03 (0.89 to 1.18)	<0.001
	2001–2011	0.70 (0.56 to 0.84)	<0.001	2011–2023	−0.59 (−0.83 to −0.35)	<0.001
	2011–2023	−1.82 (−2.01 to −1.63)	<0.001			
Rio Grande do Sul	1990–2011	−0.48 (−0.63 to −0.33)	<0.001	1990–2005	0.54 (0.25 to 0.83)	0.001
	2011–2023	−2.29 (−2.61 to −1.97)	<0.001	2005–2011	2.20 (1.84 to 2.57)	<0.001
				2011–2023	−0.3 (−0.58 to −0.01)	0.043
Santa Catarina	1990–1994	3.05 (1.14 to 5.00)	0.003	1990–2001	1.44 (1.18 to 1.70)	<0.001
	1994–2009	0.03 (−0.27 to 0.34)	0.819	2001–2023	0.29 (0.10 to 0.48)	0.004
	2009–2023	−1.98 (−2.30 to −1.65)	<0.001			
**Midwest**						
Distrito Federal	1990–2012	−0.47 (−0.57 to −0.36)	<0.001	1990–2000	1.00 (0.63 to 1.37)	<0.001
	2012–2016	−3.90 (−4.78 to −3.02)	<0.001	2000–2015	−0.44 (−0.56 to −0.33)	<0.001
	2016–2023	−2.12 (−2.90 to −1.33)	<0.001	2015–2023	−1.00 (−1.24 to −0.76)	<0.001
Goiás	1990–2009	0.78 (0.60 to 0.97)	<0.001	1990–1997	−0.39 (−1.17 to 0.40)	0.325
	2009–2013	2.29 (1.74 to 2.84)	<0.001	1997–2012	1.61 (1.38 to 1.84)	<0.001
	2013–2023	−1.50 (−1.77 to −1.22)	<0.001	2012–2023	−0.46 (−0.75 to −0.17)	0.003
Mato Grosso do Sul	1990–2016	0.22 (0.10 to 0.34)	0.001	1990–1996	3.21 (2.32 to 4.11)	<0.001
	2016–2019	−4.95 (−6.01 to −3.87)	<0.001	1996–2023	0.50 (0.36 to 0.63)	<0.001
	2019–2023	2.96 (1.57 to 4.37)	<0.001			
Mato Grosso	1990–1997	−1.26 (−2.43 to −0.08)	0.037	1990–1997	−0.78 (−1.31 to −0.26)	0.005
	1997–2002	6.73 (5.61 to 7.87)	<0.001	1997–2001	8.17 (7.17 to 9.18)	<0.001
	2002–2023	−0.75 (−1.01 to −0.48)	<0.001	2001–2023	0.33 (0.15 to 0.50)	0.001
Brazil	1990–1997	−0.16 (−0.49 to 0.17)	0.330	1990–1997	0.24 (0.01 to 0.48)	0.045
	1997–2011	0.41 (0.30 to 0.51)	<0.001	1997–2012	1.39 (1.32 to 1.47)	<0.001
	2011–2023	−1.64 (−1.76 to −1.52)	<0.001	2012–2023	−0.08 (−0.26 to 0.10)	0.377

**Source:** GBD study, 2025.

## Data Availability

All data are openly available from the Global Burden of Disease Results Tool (https://ghdx.healthdata.org/gbd-results-tool (accessed on 15 October 2025)).
